# Depth effects of trail development on herbaceous plant diversity and stress responses through flavonoid accumulation

**DOI:** 10.1007/s44154-025-00227-8

**Published:** 2025-06-09

**Authors:** Hu Su, Hu Jiang, Carly Anderson Stewart, Dina Clark, Sukuan Liu, Erin A. Manzitto-Tripp

**Affiliations:** 1 Department of Ecology and Evolutionary Biology, University of Colorado, UCB 334, Boulder, CO 80309 USA; 2 Museum of Natural History, University of Colorado, UCB 350, Boulder, CO 80309 USA

**Keywords:** Trail development, Diversity, Stress, Flavonoid, Response

## Abstract

Trail development is more prevalent as tourism develops globally. The depth effect of trail development on plant diversity and native species’ stress response via tuning flavonoids in natural ecosystems remain relatively poorly understood. We investigated the depth effects by comparing plant species diversity and flavonoid contents (of six common native species) in sampling plots plots (Rabbit Mountain Open Space, Boulder County, CO, USA) with varying distances away from trail. We found plant diversity to be lowest in plots immediately proximal to trails and highest in intermediate plots. We also found the concentrations of total flavonoids to vary significantly between plots closer and away from trails. Specifically, we found the concentrations of isoorientin and myricetin higher in plots closer to trails. On the contrary, the concentrations of vitexin and kaempferol were higher in plots away from trails. Quercetin was higher in the intermediate plots. Overall, trail development negatively impacted herbaceous plant diversity, which was evident as depth effects. The plant species responded to environmental stresses imposed by trail development through fine-tuned flavonoid accumulation.

**Supplementary Information:**

The online version contains supplementary material available at 10.1007/s44154-025-00227-8.

## Introduction

Plant community composition is a function, in part, of changing habitats along environmental gradients (Bello et al. 2013). Environmental disturbance caused by human activities have significant impacts on the diversity of native plant communities, with generally negative effects depending on mode and intensity of perturbation (Coffin 2007). These disturbances, such as coal mining (Pandey et al. 2014), road construction (Zardare et al. 2018), agricultural practices (Proulx and Mazumder 1998; Cousins 2006), and industrial outputs (Sayara et al. 2016), have all been shown to have a negative impact on the diversity of native plant communities due to altered habitats with foreign species invasion, interfered nutrients conduits, imported pollutants, and declined light conditions, among others. Although studies have very often demonstrated biodiversity loss due to increasing human activities, other studies show positive correlations between plant diversity and human activities. For instance, anthropogenic path formation (Root‐Bernstein and Svenning 2018) and total disturbance (Sahu et al. 2008) sometimes have a positive effect on the native plant diversity, the positive effects were possibly due to long‐distance linkages of propagules and nutrients, nutrient cycling, and so on. A leading theory about how environmental disturbances affect species diversity is the “intermediate disturbance hypothesis” (Connell 1978), however, recent ideas and models challenged this hypothesis and raised reasons that are not commonly recognized (Fox 2013). Overall, there were myriad, and primarily negative impacts correlations between plant diversity and human activities (Coffin 2007; Root‐Bernstein and Svenning 2018).

Environmental disturbances from human activities are also known to induce plant stress responses (Nogués-Bravo et al. 2008) due to altered community composition, hydrology, salinity, substrate compositions in the habitats (Hancock 2002; Herbert et al. 2015). Often, plant species response to these biotic and abiotic stress via production of secondary metabolites, which play a pivotal role in plant defense systems and stress responses (Akula and Ravishankar 2011; Su et al. 2015). Flavonoids are an important class of plant secondary metabolites and have been investigated extensively for their role in plant stress responses. For instance, the increased levels of flavonoids were found to be associated with drought response in *Adonis amurensis* and *A. pseudoamurens* (Gao et al. 2020). Moreover, flavonoids with ortho-dihydroxylated B-rings (e.g. catechin gallate, quercetin etc.) were shown to increase in concentration when plants were experiencing high light stress, possibly due to their reactive oxygen species scavenging and antioxidation effects (Zhang et al. 2017). Quercitol was found to increase in concentration in *Ajuga bracteosa* when cold stressed (Rani et al. 2017). In *Sorghum bicolor*, many flavonoids and flavonoid derivatives, such as naringenin, naringin, and kaempferol, were also shown to increase as a response of salt stress (Ma et al. 2020).

To depict the effects of human activities on plant diversity and the response of plant community via secondary metabolism to stresses posed by human activities, we conducted surveys and collected samples in Rabbit Mountain Open Space, a conservation area located in Boulder County, Colorado. Although prior researchers have investigated species present at this open space (Brown and Lancaster 2007; Seshadri et al. 2018), and there have been intensive studies on plant stress response through flavonoid metabolism, none have investigated the depth effects of trail development on native plant diversity at this site, and most studies of flavonoid metabolism were under controllable conditions, few focused on stress responses in natural open space.

In this study, we investigated the depth effect of disturbance caused by trail development in native plant species diversity, and plant community’ response to stresses posed by the development through flavonoid accumulation. We hypothesized that trail development would negatively impact species diversity due to altered habitats and increased biotic and abiotic stress. Additionally, we expected native species to increase flavonoids production in response to the stress posed by trail development. Elucidating how trail development affects species diversity and plant responds to anthropogenic activities through flavonoid accumulation could inform us regarding trail establishment in a manner that minimizes disturbances on native plants.

## Results

### Species diversity along the trail

We identified a total of 46 species in all study plots, spanning 21 families of flowering plants (Supplementary Table 1). These data yielded values of S, H, D and E as listed in Table 1.

**Table 1:** Indices for measuring diversity

Site	Plot	Mean S			Mean H			Mean D			Mean E		
		**0–2 m**	**2–4 m**	**4–6 m**	**0–2 m**	**2–4 m**	**4–6 m**	**0–2 m**	**2–4 m**	**4–6 m**	**0–2 m**	**2–4 m**	**4–6 m**
1	1–3	7.83^a Δ^	9.5^a*^	8.33^a^	1.37^a Δ^	1.49^a*^	1.44^a^	0.65^a Δ^	0.69^a^	0.7^a*^	0.79^a**^	0.67^a Δ^	0.79^a**^
	4–6	11.83^a^	12.17^a*^	11.33^a Δ^	1.66^A Δ^	1.8^A*^	1.77^B^	0.73^a Δ^	0.77a^**^	0.77^a**^	0.68^a Δ^	0.72^a Δ^	0.73^a*^
2	1–3	10.75^a^	11.25^a*^	10.25^a Δ^	1.43^a Δ^	1.45^a^	1.48^a*^	0.62^a Δ^	0.71^a^	0.73^a*^	0.6^a Δ Δ^	0.6^a Δ Δ^	0.64^a*^
	4–6	10.25^a*^	10^a^	8.5^a Δ^	1.21^a*^	1.04^a^	0.89^a Δ^	0.59^a*^	0.51^a^	0.42^a Δ^	0.52^a*^	0.45^a^	0.41^a Δ^
3	1–3	8^a Δ^	10.82^b*^	9^ab^	0.89^a Δ^	1.15^a*^	1.1^a^	0.44^a Δ^	0.55^a*^	0.53^a^	0.42^a Δ^	0.49^a^	0.51^a*^
	4–6	9.2^a Δ^	10.4^a*^	9.61^a^	1.28^a Δ^	1.53^a*^	1.3^a^	0.64^ab^	0.71^a*^	0.61^b Δ^	0.58^ab^	0.66^a*^	0.58^b^
4	1–3	8.4^a Δ^	8.75^ab^	10^b*^	1.47^a Δ^	1.53^ab^	1.84^b*^	0.69^A Δ^	0.7^AB^	0.8^B*^	0.72^a^	0.69^a Δ^	0.81^a*^
	4–6	8.8^a Δ^	9.8^a^	9.8^a^	1.41^a Δ^	1.67^a*^	1.55^a^	0.65^a Δ^	0.75^a*^	0.71^a^	0.65^a Δ^	0.73^a*^	0.69^a^

^*^The highest value at each side of the study sites, ^**^identical higher value, ^Δ^ the lowest value, ^Δ Δ^ identical lower value. ^a and b^ significant differences, with a *p*−value threshold set to 0.05, ^A and B^ threshold set to 0.01

Plots 1 and 4, which were immediately proximal to the trail, accounted for 62.5% (5 of 8) of the lowest S values, 87.5% (7 of 8) of the lowest H values, 75% (6 of 8) of the lowest D values, and most of the lowest E values. Intermediate distances from the trail, i.e., in plots 2 and 5, hosted the highest S values in 75%, highest H values in 62.5%, highest D values in 50% of the plots, and no lowest D values occurred at intermediate plots (Table 1). The furthest plots shared 50% (4 of 8) of the highest D values and accounted for nearly 62.6% of the highest E values (Table 1).

### Accumulation of total flavonoid with respect to distances to trail

Total flavonoid content varied as a function of plant species identity and developmental stage. The content in *Artemesia* at site 1*, Poa* at site 2, *Musineon* at site 3 and *Linaria* at site 4 decreased with an IDT. The content in *Allium* at site 4 increased with an IDT. We found no consistent trend for flavonoid content in different *Poa* collections at sites 1, 3 and 4, respectively, and in *Heterotheca* at all four sites (Supplementary Fig. 1).

**Fig. 1:**
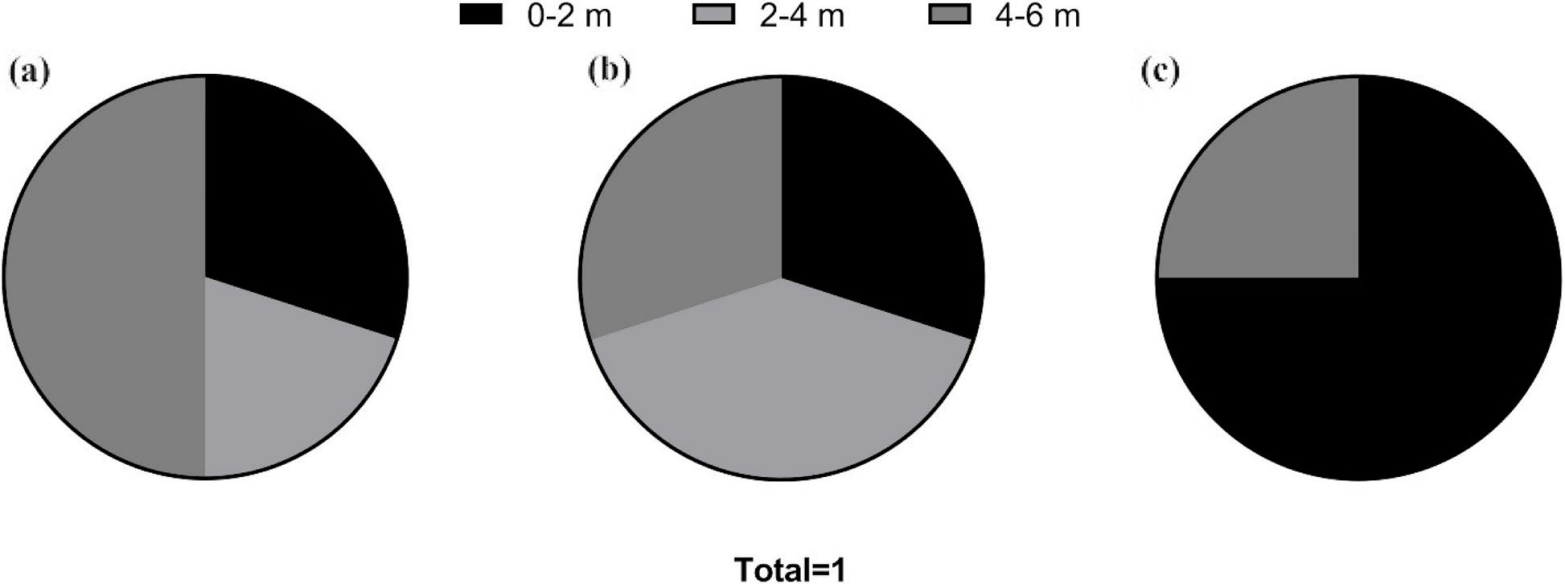
Frequency distribution of highest total flavonoid content across different plots of varying distances to the trail (0–2 m, 2–4 m, and 4–6 m). **a** early stage, **b** mid stage, and **c** late stage; note that the frequency within 2–4 m plots in late-stage collection was 0

To further explore the total flavonoid variations, we calculated the frequency of highest flavonoid content in all plots (proximal, intermediate, and furthest to trail) at different stages. We found that the proximal plot hosted a high frequency of 0.75 in LSC, the furthest plot hosted a frequency of 0.5 in ESC, and all plots hosted roughly equal frequency (proximal plots: 0.3, intermediate plots: 0.4, furthest plots: 0.3) in MSC (Fig. 1).

### Accumulation of specific flavonoid with respect to distances to the trail

Specific flavonoid contents showed different accumulations at the studied sites. For example, isoorientin in *Artemesia* at site 1 was highest in the proximal plot to the trail, whereas vitexin and quercetin peaked in the intermediate plot and kaempferol peaked in the furthest plot. Isoorientin and vitexin content in *Linaria* at site 2 increased with IDT. Isoorientin and myricetin in *Poa* at site 3 reached the highest at the intermediate plot, and quercetin and kaempferol reached the highest at the furthest plot (Supplementary Fig. 1-Fig. 7).

**Fig. 2:**
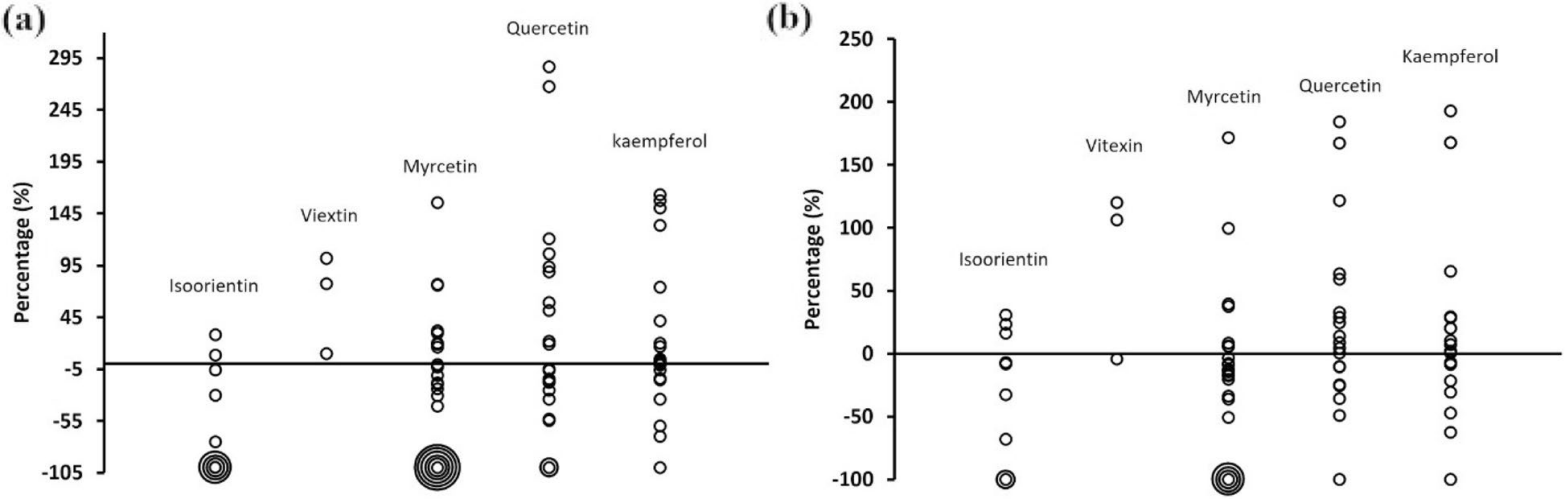
Variation of the mean of individual flavonoid content (**a**) in plots 2–4 m compared to that of plots 0–2 m, and **b** in plots 4–6 m compared to that of plots 0–2 m. Circles were used to show the overlap of data values, with one circle corresponding to data collection event

Considering the complexity of specific flavonoid accumulations, we compared mean content at distances of 2–4 m and 4–6 m to that at 0–2 m to identify trends in variations. We found that isoorientin and myricetin decreased in most collections with IDT, on the contrary, vitexin and kaempferol increased in most collections, and quercetin content show varying concentrations in intermediate plots and furthest plots compared to proximal plots (Fig. 2).

## Discussion

### Effects of trail development on plant diversity

Human activities alter components of ecosystems, affect temperature, humidity, water and light availabilities, soil properties and, in turn, impact plant species diversity. The most pervasive disturbances to biodiversity are directly or indirectly linked to road construction (Marcantonio et al. 2013). In this study, we found that trail development significantly impacted plant diversity along the Eagle Wind Trail. Plots 1 and 4, which were immediately proximal to the trail, accounted for the majority of the lowest S, H, D and E values (Table 1). In contrast, intermediate distances from the trail, i.e., in plots 2 and 5, accounted for the majority of the highest S, H and D values, and the furthest plots hosted the highest species evenness (Table 1). Taken together, our results indicate negative impacts of trail development on species diversity as a function of distance from the trail, as we hypothesized in our study.

Numerous previous studies have shown negative effects of road construction on biodiversity (T Findlay and Bourdages 2000; Baez and Balslev 2007). Our results largely concur with these prior findings. Moreover, our results indicate that these negative effects were minimized in the intermediate plots, which hosted higher diversity. Potential mechanisms for these outcomes include (1) that trail development might directly alter terrain and therefore microhabitat features (Marion and Leung 2001; Hancock 2002; Tomczyk 2011; Ballantyne and Pickering 2015), making these areas less conducive to plant survival near the trail; (2) that human and animal (horse, dog, etc.) activities damaged plants near the trail, including their reproductive potential and survival (Törn et al. 2009); and (3) that anthropogenic disturbance dispersed seeds or reproductive parts of herbs to intermediates. However, other studies have shown that areas proximal to roads or trails hosted higher diversity compared to more distant areas (Root‐Bernstein and Svenning 2018; Zardare et al. (n.d.)), and this was oftentimes attributable to the introduction of non-native species, trail types, and trail ages (Sax and Gaines 2003). Thus, taken together, there are some conflicting patterns amongst the literature, but in this study, we can conclude that trail development impacts species diversity along these trails, and there exist depth effects regarding relative distance from trail. It is important to note that there are many other factors, such as soil composites, water contents, and vegetation types, which may lead to variations in the impacts of trail development on plant community. Our study focused on Rabbit Mountain Open Space as a case study to explore the impacts of trail development in a localized context. In the future study, we should add more investigations across different regions to enhance our findings and understanding of the impacts of trail development on plant community alongside the trail.

### Total flavonoid accumulation of species at differing distances from trail

Habitat alternation as a result of human activities has been shown to elicit stress responses in plant specifically through fluctuations in secondary metabolites including flavonoid content and type (Ohtsuki et al. 2013; Sharma 2013; Meraj et al. 2020). In this study, total flavonoid content for species varied among our study sites in a nuanced manner. For example, flavonoids in *Heterotheca villosa* at site 1 and *Allium textile* at site 4 were the lowest within 0–2 m from the trail (Supplementary Fig. 1). Flavonoid content in all collections of *Artemesia ludoviciana* at site 1, *Musineon divaricatum* at site 3, in *Linaria genistifolia* at site 4, however, decreased with the IDT and were highest within 0–2 m (Supplementary Fig. 1). This supports our prediction that at distances nearest the trail, flavonoid content should be greatest. The frequency of highest total flavonoid contents in different areas indicated that highest frequency (0.75) occurred in 0–2 m PTT in LSC (Supplementary Fig. 1), supporting our hypothesis that developmental stage impacts flavonoid accumulation.

Previous studies have shown that flavonoids help mitigate stress in plants with respect to habitat conditions (Fini et al. 2011; Nakabayashi and Saito 2015; Wang et al. 2021). However, flavonoid accumulation levels nonetheless typically depend on specific plant species and stress levels (Xu et al. 2020; Gao et al. 2021). Our results indicated (1) that flavonoid accumulation might respond to stresses posted by anthropogenic activities on the Eagle Wind Trail, which was manifested as depth effects, and (2) total flavonoid accumulation trends varied with respect to species, developmental stage. These features are in agreement with previous studies. To our knowledge, there are many fewer instances of study of flavonoid accumulation in native habitats without manipulation of specific environmental factors, compared to highly controlled greenhouse conditions. As such, we suspect flavonoid metabolism is likely affected by complex cues including types of human activities, water availability, light intensity, etc., which resulted in diverse accumulation responses herein documented.

### Specific flavonoid accumulation of species at differing distances from trail

Individual flavonoid functions in response to environmental stress are varied and complex as a function of different factors investigated across studies (Nakabayashi et al. 2014; Xu et al. 2020; Alhaithloul et al. 2021). Our results indicated that individual flavonoids show diverse responses. For instance, quercetin content in *Artemesia* at site 1 peaked within intermediate distances to the trail whereas kaempferol peaked within plots furthest from the trail. Quercetin in *Heterotheca* at site 1 increased with IDT, but the trend was reversed in most samples at site 3 (Supplementary Fig. 2 and Fig. 4). Kaempferol in the early and late collections of *Linaria* at site 4 peaked within intermediate distances. In the mid-stage samples, it peaked within proximal plots (Supplementary Fig. 5). The comparison of content in species within 2–4 m and 4–6 m to that within 0–2 m showed that isoorientin and myricetin decreased with IDT in most collections (Fig. 2). This supports our hypothesis that flavonoid content should be greatest at distances nearest the trail, while vitexin and kaempferol increased in most collections. Other studies have similarly shown that a universal response with respect to flavonoid accumulation from abiotic stresses was not apparent (Cetinkaya et al. 2017), with flavonoids accumulating in differential manners. Considering the function of flavonoids as antioxidants in cells, there may be a well-coordinated antioxidant defense system in operation (Ferdinando et al. 2012), which up- or down-regulates individual flavonoid accumulations. Being like the factors affecting the plant diversity in natural ecosystem, there are also complex factors in open space that could affect the total and individual flavonoids accumulation, and we selected six plant species for flavonoid analysis based on their abundance and consistent presence along the trail gradients. Although the flavonoid accumulation of the selected species could show certain pattern, this may have the potential oversight of species that may be sensitive to environmental changes. Additionally, our findings are based on one-season sampling, this limited in-depth interpretation of our results. This provides us with a clearer direction for future research.

## Conclusions

In conclusion, our research demonstrates that there are depth effects of trail development on plant diversity and native species’ stress response via tuning flavonoids. Specifically, we identified a total of 46 species in the study area, trail development negatively affected species’ diversity, which was lowest in plots immediately proximal to trails and highest in intermediate plots. Concentrations of total flavonoids varied significantly between plots closer and away from trails. Concentrations of isoorientin and myricetin higher in plots closer to trails, vitexin and kaempferol were higher in plots away from trails, and quercetin was higher in the intermediate plots. Moreover, there are limitations in this study constrained extending our findings to broader ecosystem, there are needs for future studies to incorporate environmental variables and multi-season investigation to provide a more comprehensive understanding of the interplay between trail development and plant community.

## Materials & methods

### Study area & field sampling

**Fig. 3:**
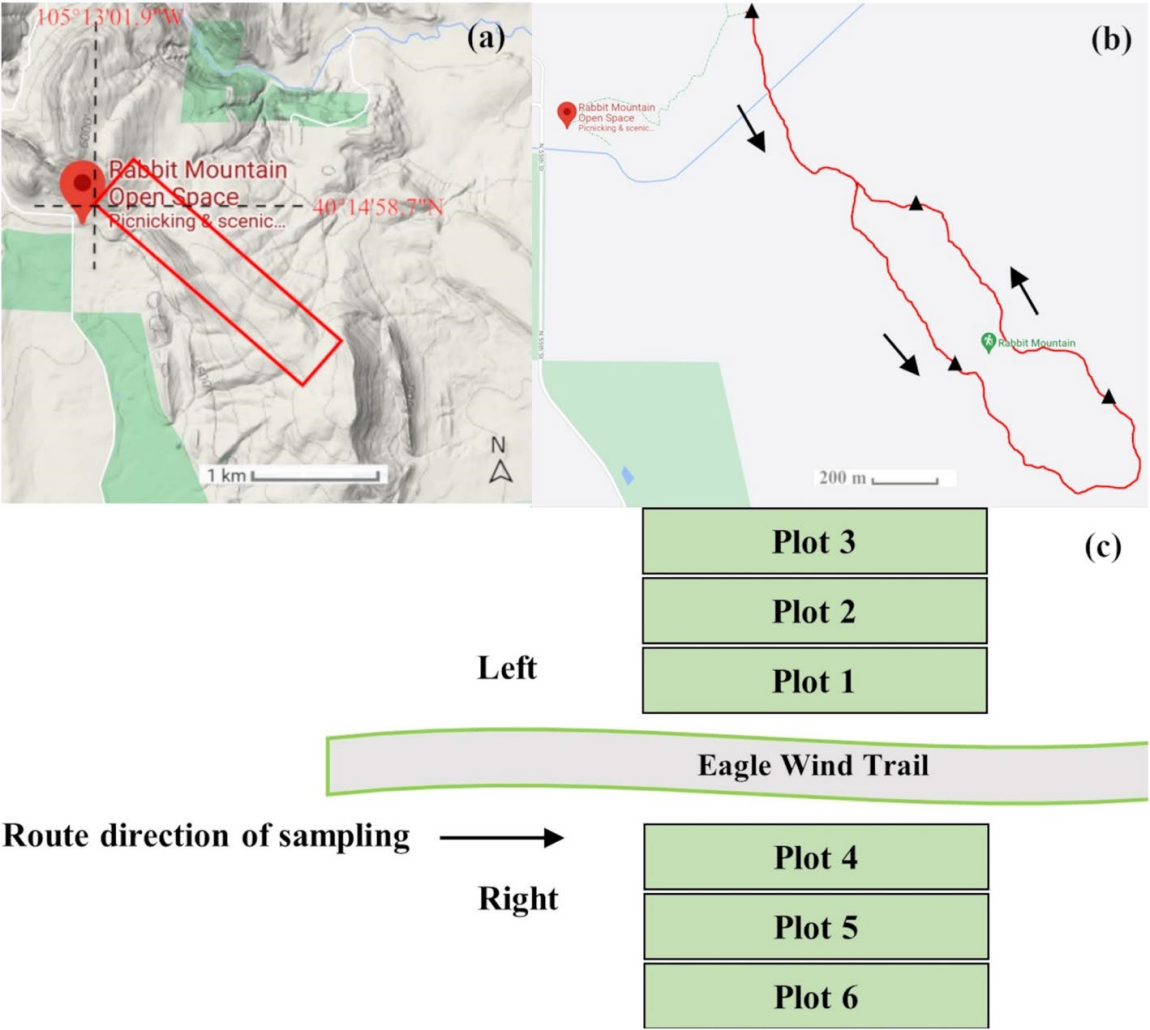
**a** Approximate study area (red rectangle) at Rabbit Mountain Open Space. **b** Eagle Wind Trail (red line), sampling sites (black triangles), and direction of sampling (black arrow). Map source: Google Maps @ 2021 Google. **c** Layout of plots at each of the four study sites, with each site hosting six plots (three on one side of the trail and three on the other). These six plots were spaced at 0–2 m (plots 1 and 4), 2–4 m (plots 2 and 5), and 4–6 m from Eagle Wind Trail (plots 3 and 6)

The study area was situated along both sides of Eagle Wind Trail located at Rabbit Mountain Open Space in Boulder County, Colorado, USA. The Eagle Wind Trail is ~ 2.5 miles long and initiates at ~ 40°14′58.7″N, 105°13′01.9″W (Fig. 3a). We sampled a total of four sites: 40°14′58.2″N, 105°13′01.2″W (site 1), 40°14′17.2″N,105°12′28.8″W (site 2), 40°14′18.5″N,105°12′12.6″W (site 3), and 40°14′39.1″N,105°12′39.6″W (site 4) (Fig. 3b). Plots 1 and 4 were within the area 0–2 m PTT, plots 2 and 5 were within 2–4 m PTT, and plots 3 and 6 were within 4–6 m PTT (Fig. 3c). The length of each plot was 80 m long × 2 m wide, arranged parallel to the trail.

At each site and plot, the total number of plant species was counted. To evaluate the effects of distance from the trail on flavonoid accumulation, we followed two criteria for collecting samples for subsequent flavonoid assay: (1) the species should present in one set of the parallel plots (i.e., it must be present in either plots 1–3 or present in plots 4–6), and (2) the number of individuals of a given species should be > 6 in each plot. Adhering to these criteria, we selected the following for flavonoid assay: *Heterotheca villosa*, *Artemesia ludoviciana* and *Poa compressa* at site 1 (plots 4–6), *Linaria genistifolia* and *Poa compressa* at site 2 (plots 1–3), *Heterotheca villosa*, *Musineon divaricatum* and *Poa compressa* at site 3 (plots 4–6), and *Linaria genistifolia*, *Allium textile* and *Poa compressa* at site 4 (plots 4–6). Samples were collected at early stage (April 27), mid stage (May 26), and late stage (July 10). Only early- and mid-stage collections were made for *Allium textile* (site 2 and 4), *Linaria genistifolia* (site 2), *Musineon divaricatum* (site 3) and *Poa compressa* (all four sites) due to seasonal growth variation and high temperatures in July at the study sites; all others were collected three times (early, mid, and late). Field-collecting methods followed standard protocols implemented by staff at the University of Colorado Herbarium (COLO) and as described by Warsh (Warsh et al. 2023) as well as Hogan (Hogan 2019). Field collections identifications were verified and/or corrected by two of us (DC and EAMT) at the COLO Herbarium, where voucher specimens representing all species present in the study plots were deposited.

### Species diversity analysis

For each plot, diversity was measured using mean value for number of species (S) (Kessler et al. 2001), Shannon–Wiener (H), Simpson (D), and Pielou (E) indices calculated following Zhu et al. (Zhu et al. 2007), as: H=$-{\sum }_{i=1}^{s}{p}_{i}$ ln ${p}_{i}$, D = 1-$\sum_{i=1}^{s}{p}_{i}^{2}$, E = $\frac{\text{H}}{\text{ln}s}$, where ${p}_{i}$ is the proportion of individuals belonging to species i (the ith species).

### Flavonoid content detection

Flavonoids were extracted following Alhaithloul et al. (Alhaithloul et al. 2021) with minor modifications. Each sample was pulverized using liquid nitrogen. We then added 85% methanol (300 µL/mg) to samples, vortexed them for 1 min, centrifuged at 15,000 rpm for 5 min, then pipetted the supernatant into a new tube. This process was repeated a second time, and the supernatants were combined.

Total flavonoid content was measured following Chang et al. (Chang et al. 2002) with minor modifications. Each 0.3 mL extraction was mixed with 0.1 mL 10% aluminum chloride, 0.1 mL 1 M potassium acetate, 1.8 mL 95% methanol, and 1.7 mL distilled water to yield a total volume of 4 mL. Mixtures were then held at room temperature for 15 min. The aluminum chloride was replaced with distilled water in a blank sample. Absorbance was measured at 415 nm using an Eppendorf Biospectrometer. Rutin was used as a reference and total flavonoid content was calculated according to a standard equation, where y = 0.0042x—0.0031 (R^2^ = 0.99, y-absorbance, x-concentration, µg/mL).

Isoorientin, vitexin, myricetin, quercetin and kaempferol are common flavonoids and accumulate in diverse plant species. We assayed concentrations of these flavonoids using an in-house HPLC (Hewlett Packard 1090 Series II system) equipped with a DAD detector, auto sample injector, and binary solvent delivery pump. We used a Merck Chromolith Performance RP-18e column (5 µm particle size, i.d. 100 mm × 4.6 mm) (Tripp et al. 2018). Extractions were filtered with a 0.22 µm nylon membrane and 10 µL of the filtrate was analyzed at a wavelength of 368 nm with a reference of 500 nm and 80 nm bandwidth.

The gradient elution program was modified according to methods implemented by Tripp et al. (2018) and Zu et al. (2006). The mobile phases were acetonitrile (A) and 2% (v/v) acetic acid (B). We used a 1 mL/min flow rate, and separation was performed at room temperature.

For *Allium textile*, *Artemesia ludoviciana*, *Heterotheca villosa* and *Linaria genistifolia*, we implemented the following program: 7% A and 93% B at 0 min, 18% A and 82% B at 10 min, 35% A and 65% B at 15 min, 17–20 min hold 55% A and 45% B, 7% A and 93% B at 22 min. For *Musineon divaricatum* and *Poa compressa*, we implemented the following: 5% A and 95% B at 0 min, 13% A and 87% B at 10 min, 20% A and 80% B at 15 min, 35% A and 65% B at 17 min, 20–24 min hold 55% A and 45% B, 5% A and 95% B at 25 min.

Quantification of each compound was conducted by creating a dilution series of a standard reference. *Allium textile*, *Artemesia ludoviciana*, *Heterotheca villosa* and *Linaria genistifolia*, the standard equations were as follows: isoorientin: y = 11.66x—0.06 (R^2^ = 0.99); vitexin: y = 15.22x—0.42 (R^2^ = 0.99); myricetin: y = 37.15x—49.3 (R^2^ = 0.99); quercetin: y = 30.7x—2.09 (R^2^ = 0.99); kaempferol: y = 36.05x—8.19 (R^2^ = 0.99), (y-peak area, x-concentration, µg/mL). For *Musineon divaricatum* and *Poa compressa*, the standard equations were as follows: isoorientin: y = 12.98x—23.34 (R^2^ = 0.99); vitexin: y = 14.41x—16.25 (R^2^ = 0.99); myricetin: y = 15.28x—5.75 (R^2^ = 0.99); quercetin: y = 82.86x—49.07 (R^2^ = 0.99); kaempferol: y = 24.31x—10.34 (R^2^ = 0.99), (y-peak area, x-concentration, µg/mL).

Variations of specific flavonoid concentration were calculated as: A (%) = (B-C)*100%/C, where A was the percentage, B was the mean specific concentration in plots 2 and 5 (2–4 m to the trail) and plots 3 and 6 (4–6 m to the trail), and C was the mean specific content in plots 1 and 4 (0–2 m to the trail).

### Data analysis

One-way ANOVA analyses were used to test the significance of changes in flavonoid content at varying distances from the trail. Analyses were conducted with Python.

## Supplementary Information

Supplementary Material 1.

## Data Availability

Data will be made available on request.

## Abbreviations

PTT: Perpendicular to the trail
IDT: Increase of distance from trail
ESC: Early-stage collection
MSC: Mid-stage collection
LSC: Late-stage collection

## References

[CR1] Akula R, Ravishankar GA (2011) Influence of abiotic stress signals on secondary metabolites in plants. Plant Signal Behav 6:1720–1731. 10.4161/psb.6.11.17613 22041989 10.4161/psb.6.11.17613 PMC3329344

[CR2] Alhaithloul HA, Galal FH, Seufi AM (2021) Effect of extreme temperature changes on phenolic, flavonoid contents and antioxidant activity of tomato seedlings (Solanum lycopersicum L.). PeerJ. 9:e11193. 10.7717/peerj.11193 34026345 10.7717/peerj.11193 PMC8123231

[CR3] Baez S, Balslev H (2007) Edge effects on palm diversity in rain forest fragments in western Ecuador. Biodivers Conserv 16:2201–2211. 10.1007/s10531-007-9159-5

[CR4] Ballantyne M, Pickering CM (2015) The impacts of trail infrastructure on vegetation and soils: Current literature and future directions. J Environ Manage 164:53–64. 10.1016/j.jenvman.2015.08.032 26342267 10.1016/j.jenvman.2015.08.032

[CR5] Brown CS, Lancaster R (2007) Establishing Native Plants on Abandoned Farmland at Rabbit Mountain Open Space, Boulder County, Colorado. https://assets.bouldercounty.gov/wp-content/uploads/2017/03/research-report-2007Brown.pdf

[CR6] Cetinkaya H, Karaman M, Karaman HS, Kulak M, Kocer F (2017) Flavonoid Accumulation Behavior in Response to the Abiotic Stress: Can a Uniform Mechanism Be Illustrated for All Plants? In: Justino G (ed) Flavonoids - From Biosynthesis to Human Health. IntechOpen, Rijeka, pp 151–165. 10.5772/68093

[CR7] Chang C-C, Yang M-H, Wen H-M, Chern J-C (2002) Estimation of total flavonoid content in propolis by two complementary colometric methods. J Food Drug Anal 10:3. 10.38212/2224-6614.2748

[CR8] Coffin AW (2007) From roadkill to road ecology: a review of the ecological effects of roads. J Transp Geogr 15:396–406. 10.1016/j.jtrangeo.2006.11.006

[CR9] Connell JH (1978) Diversity in tropical rain forests and coral reefs: high diversity of trees and corals is maintained only in a nonequilibrium state. Science 199:1302–1310. 10.1126/science.199.4335.1302 17840770 10.1126/science.199.4335.1302

[CR10] Cousins SA (2006) Plant species richness in midfield islets and road verges–the effect of landscape fragmentation. Biol Conserv 127:500–509. 10.1016/j.biocon.2005.09.009

[CR11] de Bello F, Lavorel S, Lavergne S, Albert CH, Boulangeat I, Mazel F, Thuiller W (2013) Hierarchical effects of environmental filters on the functional structure of plant communities: a case study in the French Alps. Ecography 36:393–402. 10.1111/j.1600-0587.2012.07438.x

[CR12] Ferdinando MD, Brunetti C, Fini A, Tattini M (2012) Flavonoids as Antioxidants in Plants Under Abiotic Stresses. In: Ahmad P, Prasad M (eds) Abiotic Stress Responses in Plants. Springer, New York, pp 159–179. 10.1007/978-1-4614-0634-1_9

[CR13] Findlay TCS, Bourdages J (2000) Response time of wetland biodiversity to road construction on adjacent lands. Conserv Biol 14:86–94. 10.1046/j.1523-1739.2000.99086.x

[CR14] Fini A, Brunetti C, Di Ferdinando M, Ferrini F, Tattini M (2011) Stress-induced flavonoid biosynthesis and the antioxidant machinery of plants. Plant Signal Behav 6:709–711. 10.4161/psb.6.5.15069 21448007 10.4161/psb.6.5.15069 PMC3172844

[CR15] Fox JW (2013) The intermediate disturbance hypothesis should be abandoned. Trends Ecol Evol 28:86–92. 10.1016/j.tree.2012.08.014 10.1016/j.tree.2012.08.014 22981468

[CR16] Gao G, Lv Z, Zhang G, Li J, Zhang J, He C (2021) An ABA–flavonoid relationship contributes to the differences in drought resistance between different sea buckthorn subspecies. Tree Physiol 41:744–755. 10.1093/treephys/tpaa155 33184668 10.1093/treephys/tpaa155

[CR17] Gao S, Wang Y, Yu S, Huang Y, Liu H, Chen W, He X (2020) Effects of drought stress on growth, physiology and secondary metabolites of Two Adonis species in Northeast China. Sci Hortic 259:108795. 10.1016/j.scienta.2019.108795

[CR18] Hancock PJ (2002) Human impacts on the stream–groundwater exchange zone. Environ Manage 29:763–781. 10.1007/s00267-001-0064-5 11992170 10.1007/s00267-001-0064-5

[CR19] Herbert ER, Boon P, Burgin AJ, Neubauer SC, Franklin RB, Ardón M, Hopfensperger KN, Lamers LP, Gell P (2015) A global perspective on wetland salinization: ecological consequences of a growing threat to freshwater wetlands. Ecosphere 6:1–43. 10.1890/ES14-00534.1

[CR20] Hogan T (2019) A floristic survey of the Boulder Mountain Park. J Bot Res Inst Tex 13:279–314. 10.17348/jbrit.v13.i1.852

[CR21] Kessler M, Herzog SK, Fjeldså J, Bach K (2001) Species richness and endemism of plant and bird communities along two gradients of elevation, humidity and land use in the Bolivian Andes. Divers Distrib 7:61–77. 10.1046/j.1472-4642.2001.00097.x

[CR22] Ma S, Lv L, Meng C, Zhang C, Li Y (2020) Integrative analysis of the metabolome and transcriptome of Sorghum bicolor reveals dynamic changes in flavonoids accumulation under saline–alkali stress. J Agric Food Chem 68:14781–14789. 10.1021/acs.jafc.0c06249 10.1021/acs.jafc.0c06249 33274637

[CR23] Marcantonio M, Rocchini D, Geri F, Bacaro G, Amici V (2013) Biodiversity, roads, & landscape fragmentation: Two Mediterranean cases. Appl Geogr 42:63–72. 10.1016/j.apgeog.2013.05.001

[CR24] Marion JL, Leung Y-F (2001) Trail resource impacts and an examination of alternative assessment techniques. J Park Recreat Adm 19:17–37. 10.20935/AcadEng7391

[CR25] Meraj TA, Fu J, Raza MA, Zhu C, Shen Q, Xu D, Wang Q (2020) Transcriptional factors regulate plant stress responses through mediating secondary metabolism. Genes 11:346. 10.3390/genes11040346 32218164 10.3390/genes11040346 PMC7230336

[CR26] Nakabayashi R, Mori T, Saito K (2014) Alternation of flavonoid accumulation under drought stress in *Arabidopsis thaliana*. Plant Signal Behav 9:e29518. 10.4161/psb.29518 25763629 10.4161/psb.29518 PMC4203635

[CR27] Nakabayashi R, Saito K (2015) Integrated metabolomics for abiotic stress responses in plants. Curr Opin Plant Biol 24:10–16. 10.1016/j.pbi.2015.01.003 25618839 10.1016/j.pbi.2015.01.003

[CR28] Nogués-Bravo D, Araújo M, Romdal T, Rahbek C (2008) Scale effects and human impact on the elevational species richness gradients. Nature 453:216–219. 10.1038/nature06812 18464741 10.1038/nature06812

[CR29] Ohtsuki T, Murai Y, Iwashina T, Setoguchi H (2013) Geographical differentiation inferred from flavonoid content between coastal and freshwater populations of the coastal plant Lathyrus japonicus (Fabaceae). Biochem Syst Ecol 51:243–250. 10.1016/j.bse.2013.09.004

[CR30] Pandey B, Agrawal M, Singh S (2014) Coal mining activities change plant community structure due to air pollution and soil degradation. Ecotoxicology 23:1474–1483. 10.1007/s10646-014-1289-4 25017960 10.1007/s10646-014-1289-4

[CR31] Proulx M, Mazumder A (1998) Reversal of grazing impact on plant species richness in nutrient-poor vs. nutrient-rich ecosystems. Ecology 79:2581–2592. 10.2307/176502

[CR32] Rani R, Khan MA, Kayani WK, Ullah S, Naeem I, Mirza B (2017) Metabolic signatures altered by in vitro temperature stress in Ajuga bracteosa Wall. ex. Benth Acta Physiol Plant 39:1–10. 10.1007/s11738-017-2394-9

[CR33] Root-Bernstein M, Svenning J (2018) Human paths have positive impacts on plant richness and diversity: A meta-analysis. Ecol Evol 8:11111–11121. 10.1002/ece3.4578 30519429 10.1002/ece3.4578 PMC6262937

[CR34] Sahu P, Sagar R, Singh J (2008) Tropical forest structure and diversity in relation to altitude and disturbance in a Biosphere Reserve in central India. Appl Veg Sci 11:461–470. 10.3170/2008-7-18537

[CR35] Sax DF, Gaines SD (2003) Species diversity: from global decreases to local increases. Trends Ecol Evol 18:561–566. 10.1016/S0169-5347(03)00224-6

[CR36] Sayara T, Hamdan Y, Basheer-Salimia R (2016) Impact of Air Pollution from Quarrying and Stone Cutting Industries on Agriculture and Plant Biodiversity. Resour Environ 6:122–126. 10.5923/j.re.20160606.04

[CR37] Seshadri A, Hardin J, Sauer S (2018) Bringing back flowering plants and pollinators through effective control of invasive winter annual grasses with Esplanade® herbicide. Boulder City Open Space Small Grant Report Boulder CO, https://assets.bouldercounty.gov/wp-content/uploads/2019/03/bringing-back-flowering-plants-pollinators.pdf

[CR38] Sharma P (2013) Salicylic acid: a novel plant growth regulator–role in physiological processes and abiotic stresses under changing environments. In: Tuteja N, Gill SS (eds) Climate Change and Plant Abiotic Stress Tolerance. Wiley, Weinheim, pp 939–990. 10.1002/9783527675265.ch36

[CR39] Su H, Jiang H, Li Y (2015) Effects of PAL and ICS on the production of total flavonoids, daidzein and puerarin in Pueraria thomsonii Benth. suspension cultures under low light stress. J Plant Biochem Biotechnol 24:34–41. 10.1007/s13562-013-0233-7

[CR40] Tomczyk AM (2011) A GIS assessment and modelling of environmental sensitivity of recreational trails: The case of Gorce National Park, Poland. Appl Geogr 31:339–351. 10.1016/j.apgeog.2010.07.006

[CR41] Törn A, Tolvanen A, Norokorpi Y, Tervo R, Siikamäki P (2009) Comparing the impacts of hiking, skiing and horse riding on trail and vegetation in different types of forest. J Environ Manage 90:1427–1434. 10.1016/j.jenvman.2008.08.014 18930578 10.1016/j.jenvman.2008.08.014

[CR42] Tripp EA, Zhuang Y, Schreiber M, Stone H, Berardi AE (2018) Evolutionary and ecological drivers of plant flavonoids across a large latitudinal gradient. Mol Phylogenet Evol 128:147–161. 10.1016/j.ympev.2018.07.004 30017824 10.1016/j.ympev.2018.07.004

[CR43] Wang M, Zhang Y, Zhu C, Yao X, Zheng Z, Tian Z, Cai X (2021) EkFLS overexpression promotes flavonoid accumulation and abiotic stress tolerance in plant. Physiol Plant 172:1966–1982. 10.1111/ppl.13407 33774830 10.1111/ppl.13407

[CR44] Warsh S, de Silva I, Manzitto-Tripp E (2023) A Floristic Inventory of Two Boulder County Open Space Parcels: Heil Valley Ranch and Hall Ranch, Colorado, U.S.A. Madroño 69. 10.3120/0024-9637-69.3.263

[CR45] Xu Z, Zhou J, Ren T, Du H, Liu H, Li Y, Zhang C (2020) Salt stress decreases seedling growth and development but increases quercetin and kaempferol content in Apocynum venetum. Plant Biol 22:813–821. 10.1111/plb.13128 32378758 10.1111/plb.13128

[CR46] Zardare YZ, Chay MMF, Alizadeh A, Motazeh AG (2018) Assessment of Road Construction Effects on Biodiversity and Forest Composition. J Biochem Technol 9:21–29

[CR47] Zhang Q, Liu M, Ruan J (2017) Metabolomics analysis reveals the metabolic and functional roles of flavonoids in light-sensitive tea leaves. BMC Plant Biol 17:1–10. 10.1186/s12870-017-1012-8 28320327 10.1186/s12870-017-1012-8 PMC5359985

[CR48] Zhu J, Mao Z, Hu L, Zhang J (2007) Plant diversity of secondary forests in response to anthropogenic disturbance levels in montane regions of northeastern China. J for Res 12:403–416. 10.1007/s10310-007-0033-9

[CR49] Zu Y, Li C, Fu Y, Zhao C (2006) Simultaneous determination of catechin, rutin, quercetin kaempferol and isorhamnetin in the extract of sea buckthorn (Hippophae rhamnoides L.) leaves by RP-HPLC with DAD. J Pharm Biomed Anal 41:714–719. 10.1016/j.jpba.2005.04.052 10.1016/j.jpba.2005.04.052 16520013

