# Supplementary material for: Depth effects of trail development on herbaceous plant diversity and stress responses through flavonoid accumulation
Source: Stress Biol. 2025 Jun 9;5(1):40. doi: 10.1007/s44154-025-00227-8 (PMC12146240; doi:10.1007/s44154-025-00227-8)
Supplement: Supplementary file 1 — Supplementary Material 1. [file 44154_2025_227_MOESM1_ESM.docx]

**Depth effects of trail development on herbaceous plant diversity and stress responses through flavonoid accumulation**

**Stress Biology**

Hu Su^1,2*^, Hu Jiang^1,2^, Carly Anderson Stewart^1^, Dina Clark^2^, Sukuan Liu^1^, Erin A. Manzitto-Tripp^1, 2^

**Affiliation:**

^1^ Department of Ecology and Evolutionary Biology, University of Colorado, UCB 334, Boulder, CO 80309, USA

^2^ Museum of Natural History, University of Colorado, UCB 350, Boulder, CO 80309, USA

***Corresponding author:** Hu Su, e-mail: [husu.boulder@outlook.com](mailto:husu.boulder@outlook.com)

**Supplementary materials**

Supplementary Table 1 Identified species

| **No.** | **Scientific name** | **Family** |
| --- | --- | --- |
| 1 | *Alyssum simplex* | *Brassicaceae* |
| 2 | *Allium textile* | *Amaryllidaceae* |
| 3 | *Ambrosia psilostachya* | *Asteraceae* |
| 4 | *Artemesia ludoviciana* | *Asteraceae* |
| 5 | *Astragalus drummondii* | *Fabaceae* |
| 6 | *Bassia sieversiana* | *Chenopodiaceae* |
| 7 | *Bromus sp.* | *Poaceae* |
| 8 | *Calylophus serrulatus* | *Onagraceae* |
| 9 | *Cf. senecio* | *Asteraceae* |
| 10 | *Chenopodium sp.* | *Chenopodiaceae* |
| 11 | *Cirsium undulatum* | *Asteraceae* |
| 12 | *Claytonia caroliniana* | *Montiaceae* |
| 13 | *Convolvulus arvensis* | *Convolvulaceae* |
| 14 | *Dalea purpurea* | *Fabaceae* |
| 15 | *Delphinium carolinianum* | *Ranunculaceae* |
| 16 | *Dichanthelium wilcoxianum* | *Poaceae* |
| 17 | *Erigeron cf. divergens* | *Asteraceae* |
| 18 | *Eriogonum umbellatum* | *Polygonaceae* |
| 19 | *Erodium cicutarium* | *Geraniaceae* |
| 20 | *Euphorbia cf. esula* | *Euphorbiaceae* |
| 21 | *Evolvulus nuttallianus* | *Convolvulaceae* |
| 22 | *Drymocallis fissa* | *Rosaceae* |
| 23 | *Gaillardia aristata* | *Asteraceae* |
| 24 | *Gaura coccinea* | *Onagraceae* |
| 25 | *Geranium maculatum* | *Geraniaceae* |
| 26 | *Helianthus pumilus* | *Asteraceae* |
| 27 | *Heterotheca villosa* | *Asteraceae* |
| 28 | *Leucocrinum montanum* | *Asparagaceae* |
| 29 | *Linaria genistifolia* | *Plantaginaceae* |
| 30 | *Musineon divaricatum* | *Apiaceae* |
| 31 | *Oenothera Howardii* | *Onagraceae* |
| 32 | *Oenothera sp.* | *Onagraceae* |
| 33 | *Opuntia macrorhiza* | *Cactaceae* |
| 34 | *Oxybaphus linearis* | *Nyctaginaceae* |
| 35 | *Phyla cuneifolia* | *Verbenaceae* |
| 36 | *Poa compressa* | *Poaceae* |
| 37 | *Ratibida columnifera* | *Asteraceae* |
| 38 | *Rosa arkansana* | *Rosaceae* |
| 39 | *Rosa woodsii* | *Rosaceae* |
| 40 | *Scutellaria brittonii* | *Lamiaceae* |
| 41 | *Sphaeralcea coccinea* | *Malvaceae* |
| 42 | *Talinum parviflorum* | *Montiaceae* |
| 43 | *Taraxacum officinale* | *Asteraceae* |
| 44 | *Tragopogon dubius* | *Asteraceae* |
| 45 | *Unknown fabaceae* | *Unknown fabaceae* |
| 46 | *Psoralidium tenuiflorum* | *Fabaceae* |


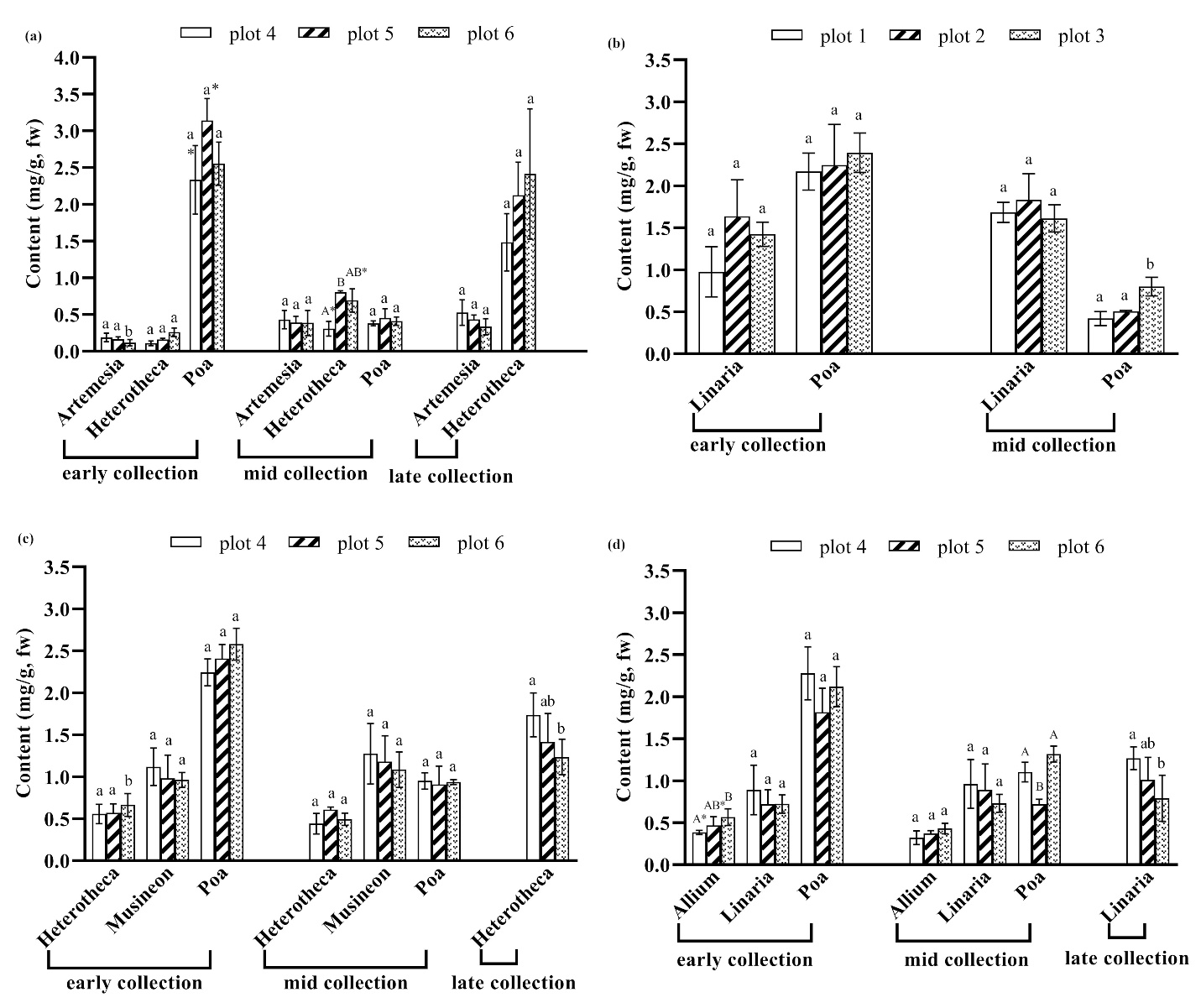


**Supplementary Fig. 1** Total flavonoid content at: (a) site 1 in *Artemesia ludoviciana* (n=9), *Heterotheca villosa* (n=9) and *Poa compressa* (n=12), (b) site 2 in *Linaria genistifolia* (n=8) and *Poa compressa* (n=12) , (c) site 3 in *Heterotheca villosa* (n=7), *Musineon divaricatum* (n=9) and *Poa compressa* (n=15), (d) site 4 in *Allium textile* (n=8), *Linaria genistifolia* (n=8) and *Poa compressa* (n=15) (a and b: significant differences, with p-value threshold set to 0.05, A and B: P-value threshold set to 0.01, * P=0.05)


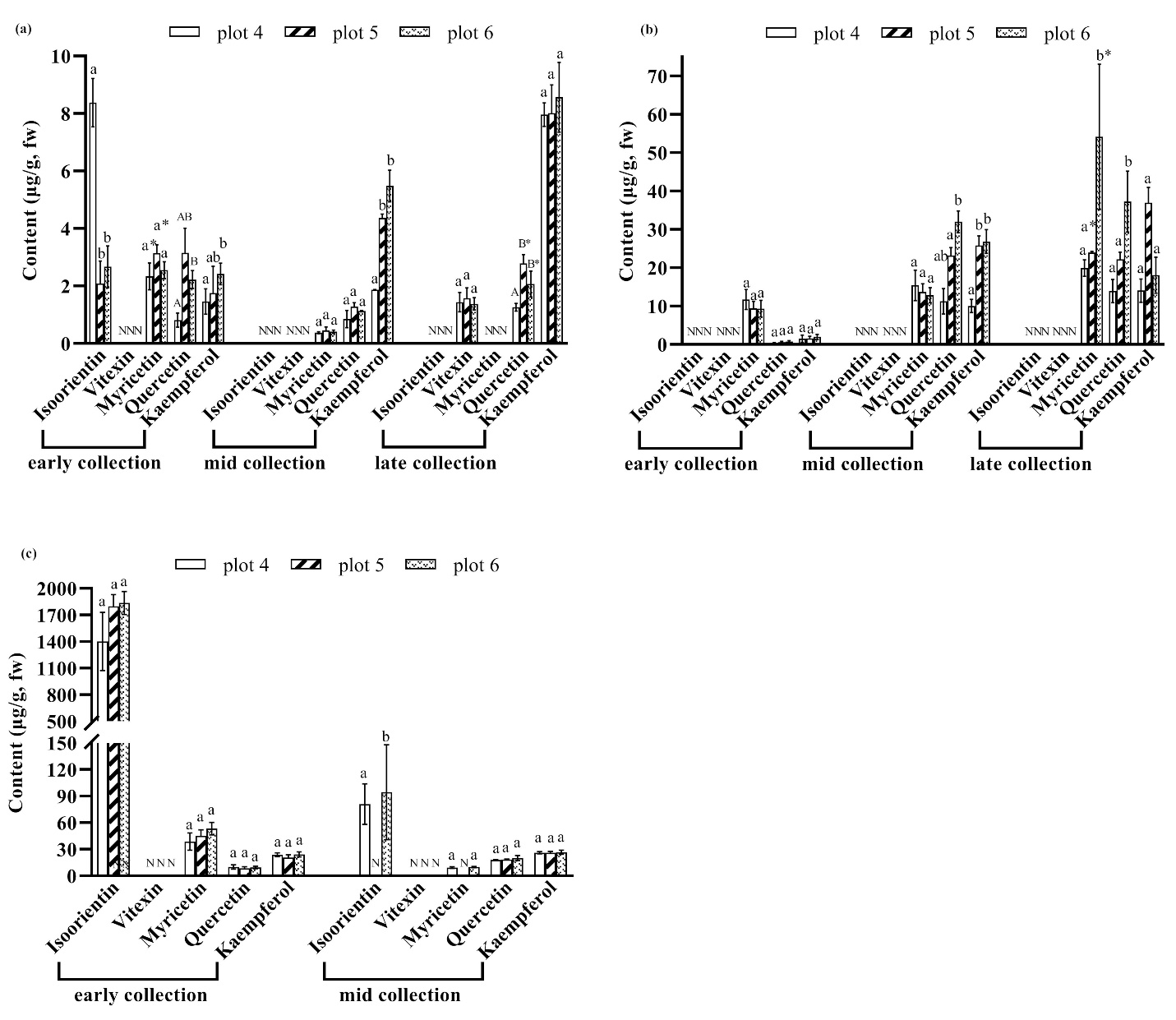


**Supplementary Fig. 2**  Specific flavonoid content in (a) *Artemesia ludoviciana* (n=9), (b) *Heterotheca villosa* (n=9) and (c) *Poa compressa* (n=12) at site 1 (N-none detected, a and b: significant differences, with p-value threshold set to 0.05, A and B: P-value threshold set to 0.01, * P=0.05)


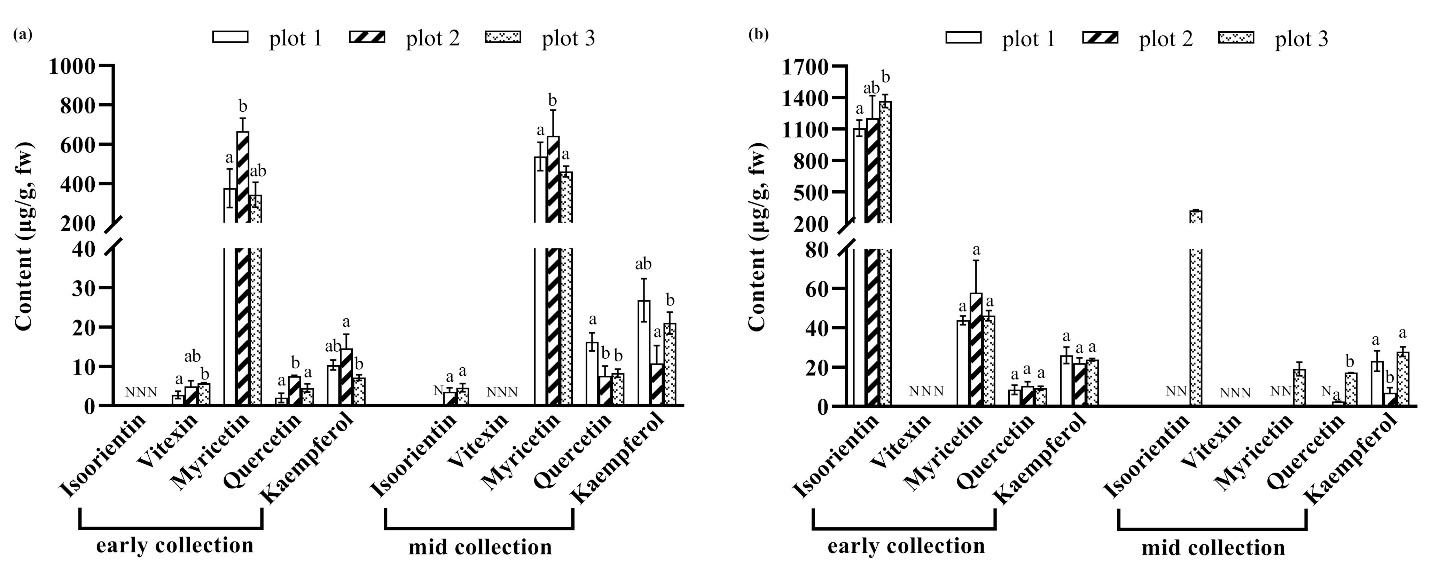


**Supplementary Fig. 3** Specific flavonoid in (a) *Linaria genistifolia* (n=7) and (b) *Poa compressa* (n=12) at site 2 (N-none detected, a and b: significant differences, with P-value threshold set to 0.05)


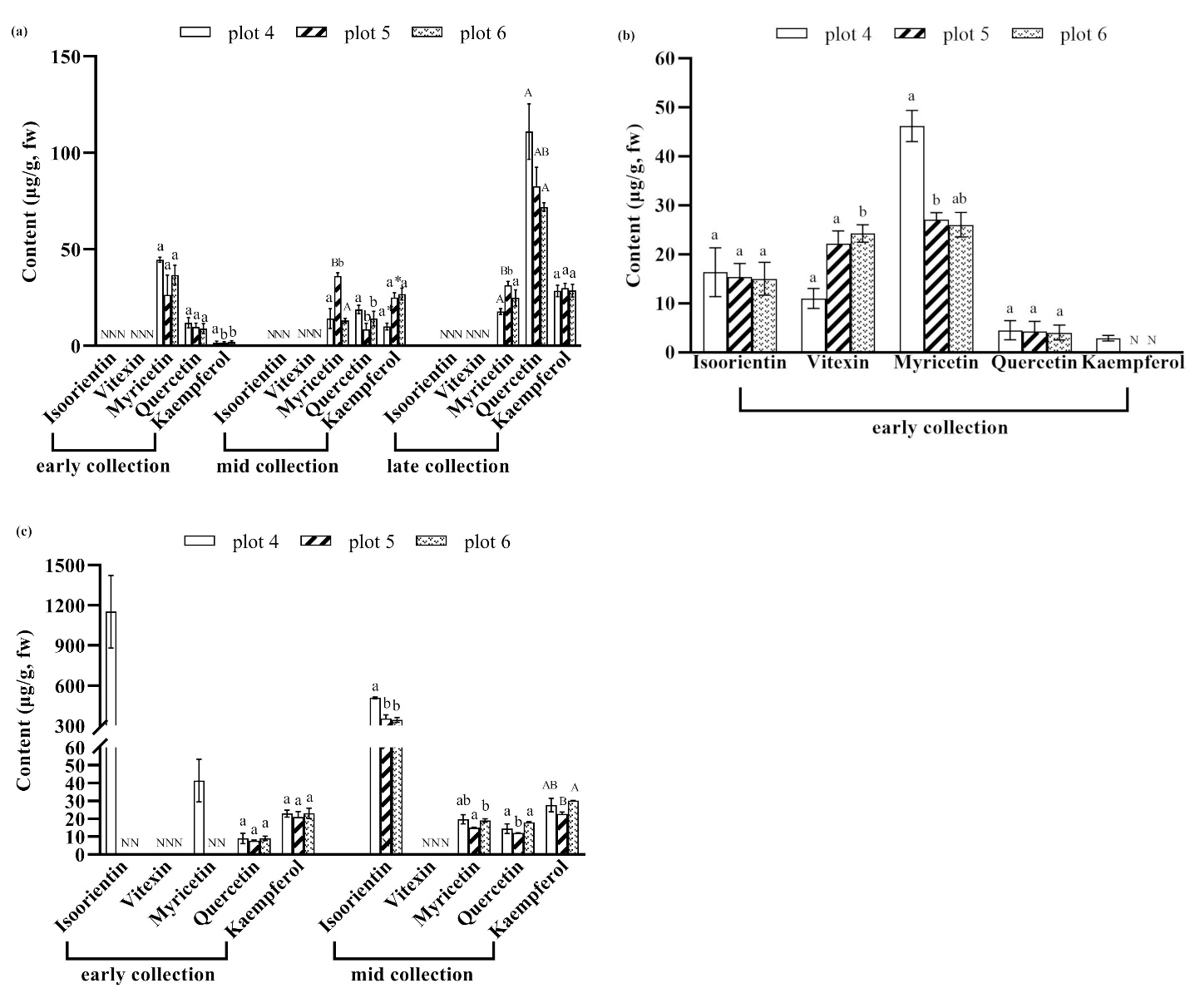


**Supplementary Fig. 4** Specific flavonoid content in (a) *Heterotheca villosa* (n=9), (b) *Musineon divaricatum* (n=8) and (c) *Poa compressa* (n=15) at site 3 (N-none detected, a and b: significant differences, with p-value threshold set to 0.05, A and B: P-value threshold set to 0.01, * P=0.05)


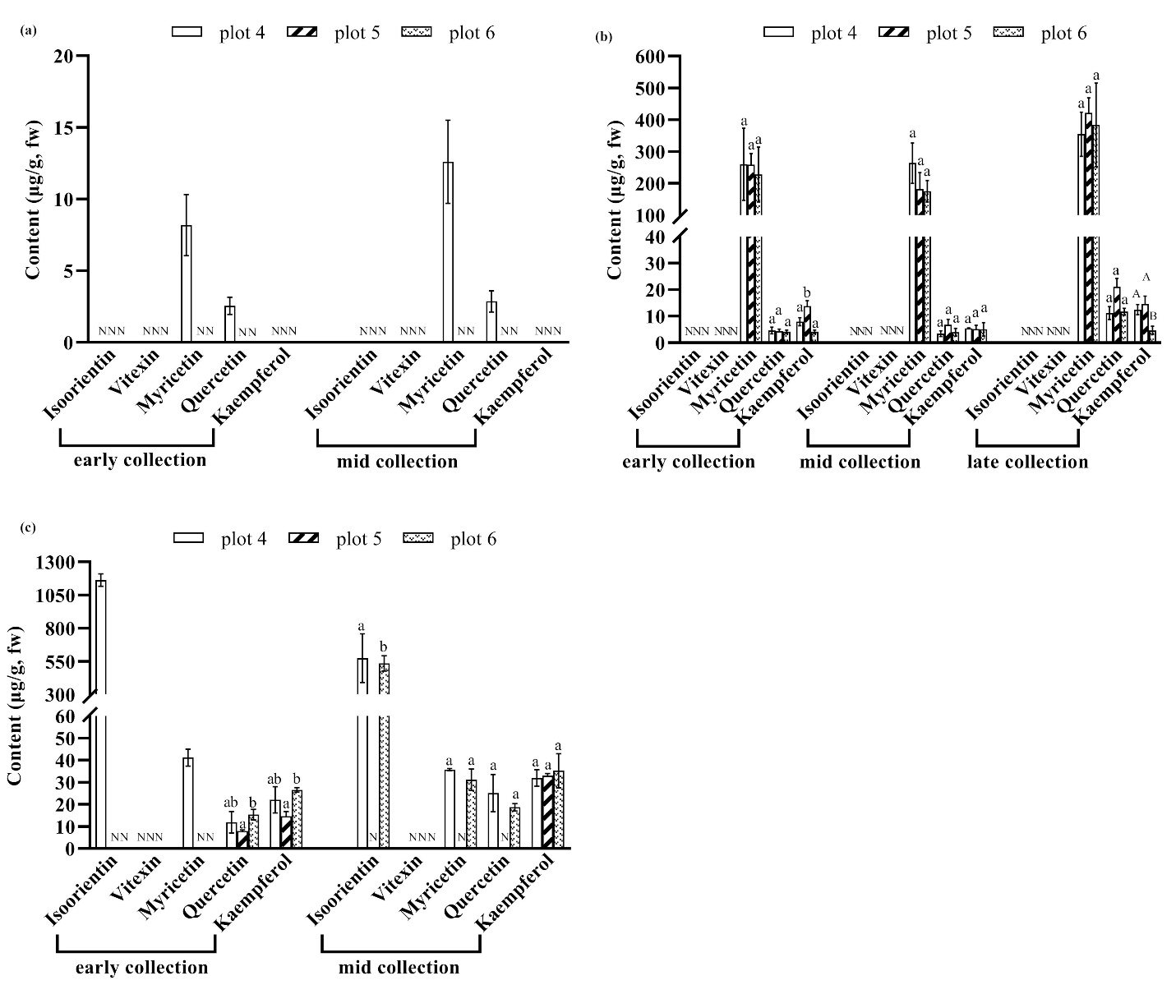


**Supplementary Fig. 5** Specific flavonoid content in (a) *Allium textile* (n=6), (b) *Linaria genistifolia* (n=7) and (c) *Poa compressa* (n=15) at site 4 (N-none detected, a and b: significant differences, with p-value threshold set to 0.05, A and B: P-value threshold set to 0.01)

**
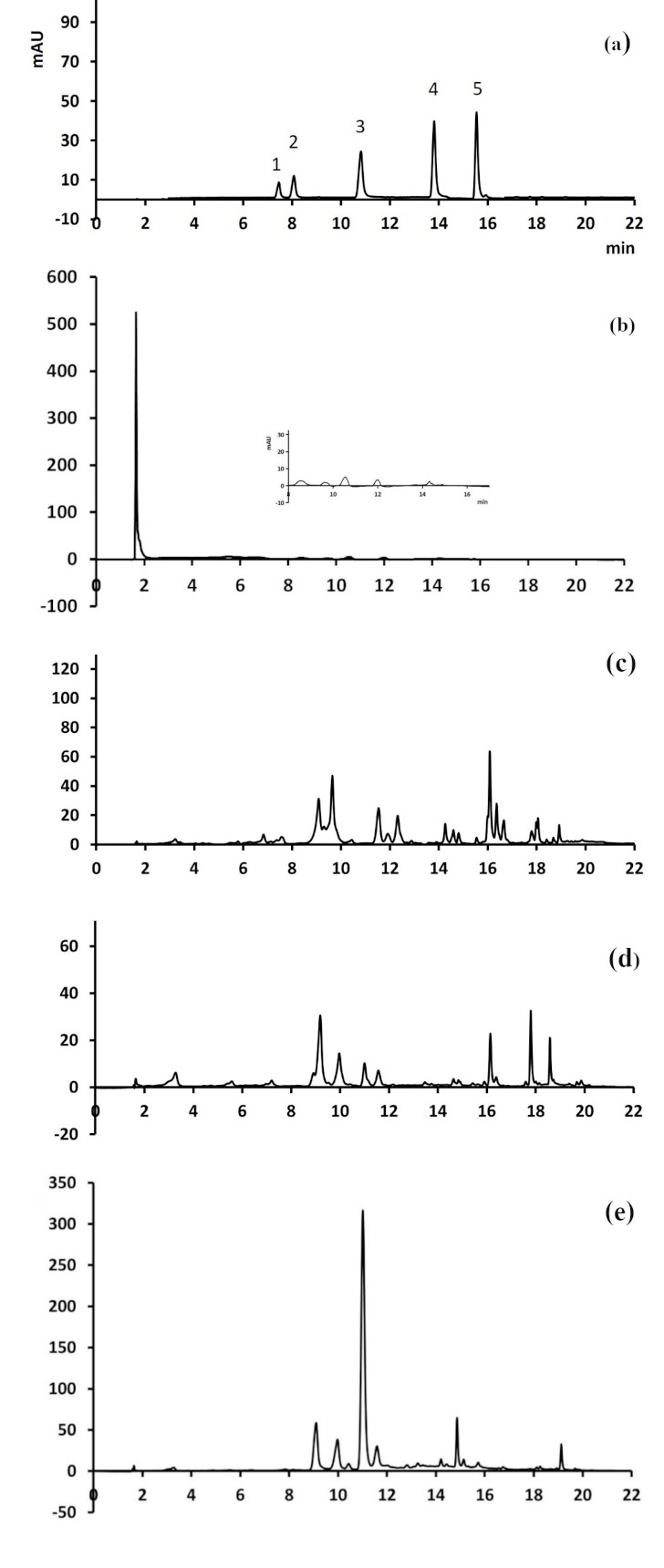
**

**Supplementary Fig. 6** Chromatograms of (a) standard reference, (b) *Allium textile*, (c) *Artemesia ludoviciana**, (d) *Heterotheca villosa*, (e) *Linaria genistifolia* extraction. (Peak 1-Isoorientin, 2-Vitexin, 3-Myricetin, 4-Quercetin, 5-Kaempferol, *-early sample)


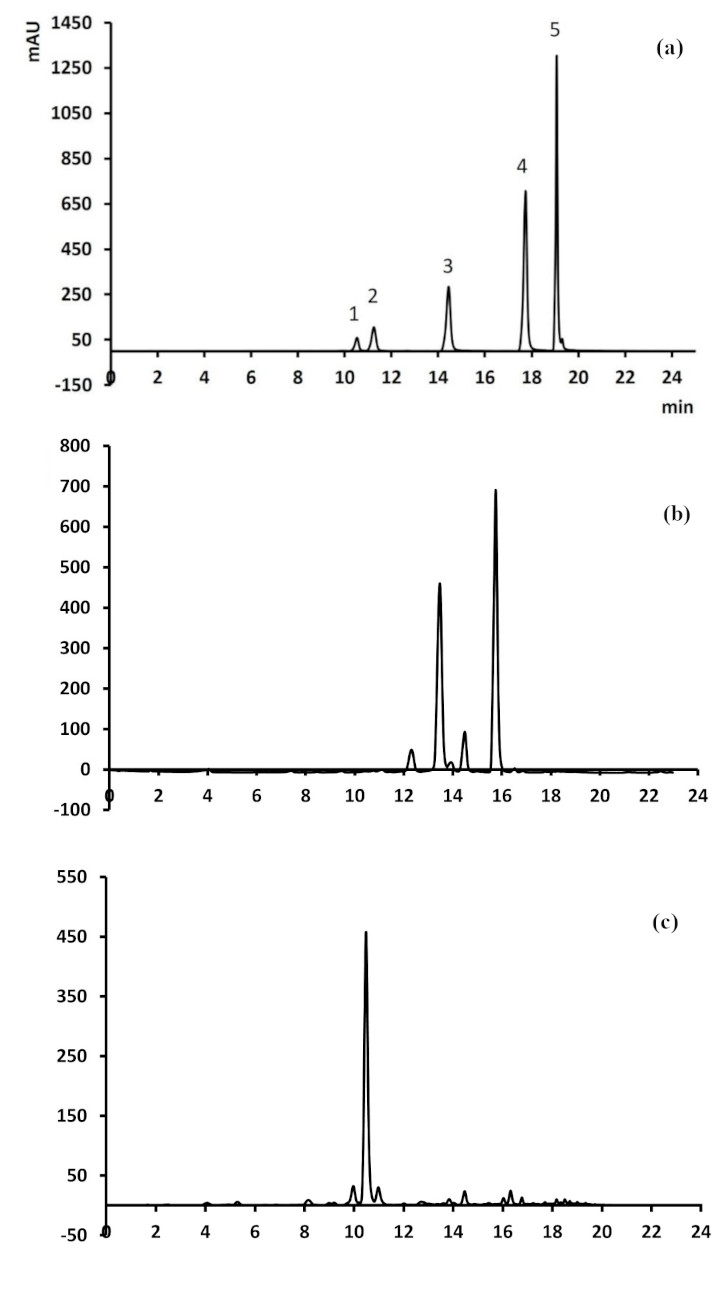


**Supplementary Fig. 7** Chromatograms of (a) standard reference, (b) *Musineon divaricatum*, (c) *Poa compressa* extraction. (Peak 1-Isoorientin, 2-Vitexin, 3-Myricetin, 4-Quercetin, 5-Kaempferol)
